# Characterization and Bioactivity Evaluation of (Polyetheretherketone/Polyglycolicacid)-Hydroyapatite Scaffolds for Tissue Regeneration

**DOI:** 10.3390/ma9110934

**Published:** 2016-11-18

**Authors:** Cijun Shuai, Chenying Shuai, Ping Wu, Fulai Yuan, Pei Feng, Youwen Yang, Wang Guo, Xiaohan Fan, Ting Su, Shuping Peng, Chengde Gao

**Affiliations:** 1 State Key Laboratory of High Performance Complex Manufacturing, the State Key Laboratory for Powder Metallurgy, Central South University, Changsha 410083, China; shuai@csu.edu.cn (Ci.S.); shuaichenying@csu.edu.cn (Ch.S.); fengpei@csu.edu.cn (P.F.); yangyouwen@csu.edu.cn (Y.Y.); guowang@csu.edu.cn (W.G.); 2 State Key Laboratory of Solidification Processing, Northwestern Polytechnical University, Xi’an 710072, China; 3 College of Chemistry, Xiangtan University, Xiangtan 411105, China; pingwu@xtu.edu.cn; 4 Hunan Farsoon High-Technology Co. Ltd., Changsha 410205, China; fulaiyuan2010@163.com; 5 Health Management Center, Xiangya Hospital, Central South University, Changsha 410008, China; fanxiaohan@farsoon.com (X.F.); suting@farsoon.com (S.T.); 6 The Key Laboratory of Carcinogenesis of the Chinese Ministry of Health, Xiangya Hospital, Central South University, Changsha 410008, China; 7 The Key Laboratory of Carcinogenesis and Cancer Invasion of the Chinese Ministry of Education, Cancer Research Institute, Central South University, Changsha 410078, China; 8 Hunan Key Laboratory of Nonresolving Inflammation and Cancer, Disease Genome Research Center, The Third Xiangya Hospital, Central South University, Changsha 410078, China

**Keywords:** bioactivity, biocompatibility, Hydroxyapatite, composite scaffolds, selective laser sintering

## Abstract

Bioactivity and biocompatibility are crucial for tissue engineering scaffolds. In this study, hydroxyapatite (HAP) was incorporated into polyetheretherketone/polyglycolicacid (PEEK/PGA) hybrid to improve its biological properties, and the composite scaffolds were developed via selective laser sintering (SLS). The effects of HAP on physical and chemical properties of the composite scaffolds were investigated. The results demonstrated that HAP particles were distributed evenly in PEEK/PGA matrix when its content was no more than 10 wt %. Furthermore, the apatite-forming ability became better with increasing HAP content after immersing in simulated body fluid (SBF). Meanwhile, the composite scaffolds presented a greater degree of cell attachment and proliferation than PEEK/PGA scaffolds. These results highlighted the potential of (PEEK/PGA)-HAP scaffolds for tissue regeneration.

## 1. Introduction

Polyetheretherketone (PEEK), as an attractive scaffold biomaterial, possesses excellent mechanical characteristics, great thermal stability, as well as good processability [1,2,3], while bioinertness and slow degradability inhibit its further application [4,5]. Polyglycolicacid (PGA) has gained much attention due to its remarkable hydrophilicity and degradability [6,7]. Combination of the advantages of PEEK and PGA could realize the expected properties better than to one of them alone. More importantly, the PEEK/PGA hybrid is able to degrade gradually, and the degradation rates could be tuned by adjusting PGA content. However, its unsolved problem is a lack of the natural cell recognition sites [8,9,10].

Hydroxyapatite (HAP, Ca_10_(PO_4_)_6_(OH)_2_), the main inorganic component of bone, has caused considerable interest for bone substitution and regeneration, owing to its strong osteoconductive properties, bone-bonding ability, preeminent biocompatibility and bioactivity [11,12,13]. Recently, Lee et al. prepared nanofibrous PLGA/Gelatin/HAP scaffolds via an electrospinning method and found that the scaffolds exhibited improved biocompatibility and osteoconductivity compared with the PLGA/Gelatin scaffolds [14]. Zhang et al. fabricated poly(l-lactide)/poly(lactide-coglycolide)/HAP scaffolds by high-pressure compression molding plus salt-leaching method and discovered that adding HAP into the scaffolds led to an encouraging improvement in bioactivity [15]. Raafat et al. developed HAP-(starch/*N*-vinylpyrrolidone) composites using γ-radiation-induced graft copolymerization and confirmed that these composites were both bioactive and biocompatible [16]. 

In this study, incorporation of HAP into PEEK/PGA hybrid has laid a particular basis for enhancing the scaffold biocompatibility and bioactivity. (PEEK/PGA)-HAP scaffolds containing different content of HAP were prepared via selective laser sintering (SLS) systems. The composite scaffolds were assessed though X-ray diffraction (XRD), scanning electron microscope (SEM), differential scanning calorimetry (DSC) and infrared spectroscopy (FTIR). The effects of HAP on apatite-forming ability were evaluated after soaking into simulated body fluid (SBF). The mechanical characteristics and MG-63 cells responses of scaffolds were investigated.

## 2. Results and Discussion

### 2.1. Scaffold Fabrication

**Figure 1:**
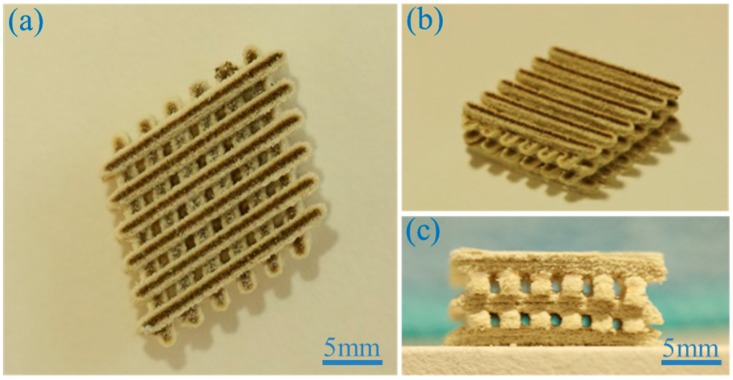
(**a**) Top view; (**b**) isometric view; and (**c**) lateral view of the polyetheretherketone/ polyglycolicacid-hydroxyapatite (PEEK/PGA-HAP) composite scaffold.

The scaffolds were developed using PEEK/PGA-HAP composite powders with different HAP content (0, 5, 7.5, 10, 12.5 and 15 wt %) through SLS. The composite scaffold was displayed from different views (Figure 1). Its overall dimension was about 18 × 18 × 7.5 mm^3^. The scaffold was made up of six struts, and each layer was 1.1 mm in width and 1.3 mm in thickness. A uniform porous and interconnected structure was presented, which was conducive to cellular growth, proliferation and nutrient transportation. The porosity of the scaffold with the optimal mechanical properties was evaluated. The apparent volume was 2430 mm^3^ based on geometry (*V_p_* = L × W × H = 18 mm × 18 mm × 7.5 mm). The actual volume was 1241 mm3 (*V_a_*) based on the Archimedes method. Its actual porosity was 48.9% ± 1.3%. The shape of the pore was square, and pore size was about 800–900 μm. High porosity and suitable pore size could enhance cell attachment and proliferation inside scaffolds, vascularization, as well as bone growth [17].

### 2.2. Microstructure and Composition

**Figure 2:**
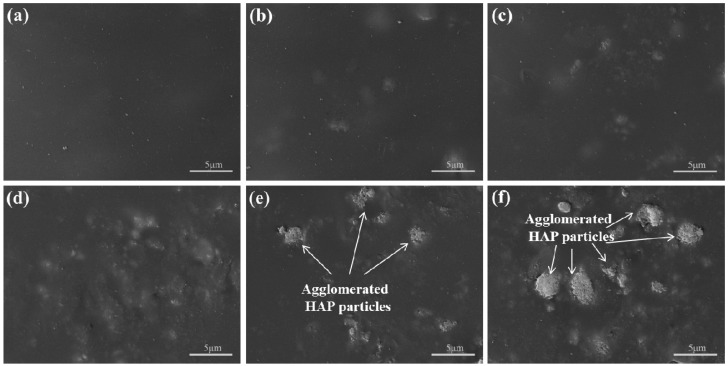
Morphologies of the scaffolds with (**a**) 0 wt %; (**b**) 5 wt %; (**c**) 7.5 wt %; (**d**) 10 wt %; (**e**) 12.5 wt %; (**f**) 15 wt % HAP.

The surface structure of scaffolds with different HAP contents (Figure 2) was presented to study the dispersed state of HAP powders in the PEEK/PGA matrix. The PEEK/PGA scaffold showed a smooth surface (Figure 2a). The even dispersion of HAP particles in PEEK/PGA matrix was observed in the scaffolds containing 5, 7.5 and 10 wt % HAP and the agglomerations of HAP particles was negligible (Figure 2b–d). In contrast, the scaffold with 12.5 wt % and 15 wt % HAP yielded significantly agglomeration and the particles were non-evenly distributed in the PEEK/PGA matrix (Figure 2e,f).

**Figure 3:**
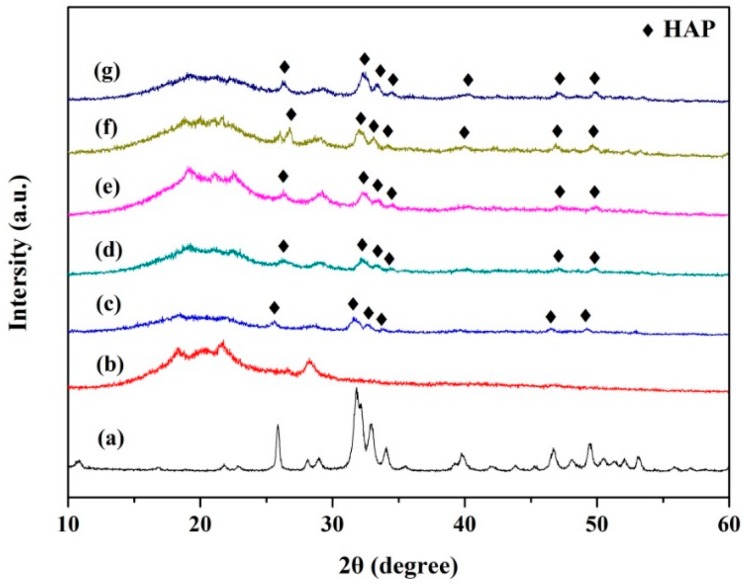
X-ray diffraction (XRD) patterns of (**a**) the HAP powder; (**b**) the PEEK/PGA scaffold; (**c**) the PEEK/PGA-5 wt % HAP scaffold; (**d**) the PEEK/PGA-7.5 wt % HAP scaffold; (**e**) the PEEK/PGA-10 wt % HAP scaffold; (**f**) the PEEK/PGA-12.5 wt % HAP scaffold and (**g**) the PEEK/PGA-15 wt %.

The phase compositions of the scaffolds containing different content of HAP were determined and graphed in XRD spectra (Figure 3). PEEK phase and PGA phase, as the predominant components of the scaffolds, were clearly shown in all the patterns. For the scaffolds with 5, 7.5, 10, 12.5 and 15 wt % HAP, their patterns exhibited some peaks at 2θ = 31.8, 32.9, 34.3, 46.7 and 49.5, which correspond to (211), (300), (202), (222) and (213) characteristic spectrum of HAP (Figure 3c–g), respectively [18]. Meanwhile, with the increase of HAP, these peaks increased in intensity. In addition, no extra peaks of other phases were observed.

### 2.3. DSC Studies

**Figure 4:**
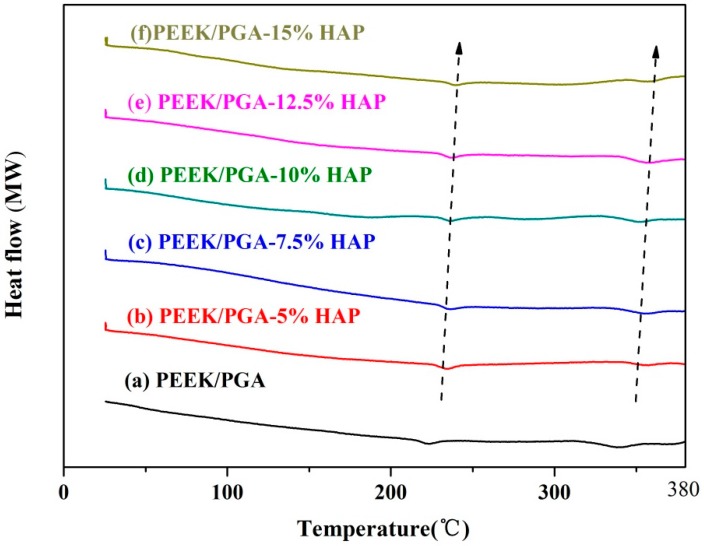
Differential scanning calorimetry (DSC) spectra of PEEK/PGA scaffold and PEEK/PGA-HAP composite scaffolds.

DSC was conducted to detect the physical states of HAP particles within composite scaffolds (Figure 4). The DSC thermogram of PEEK/PGA scaffold showed two distinct endothermic peaks at approximately 343 °C and 224 °C, which was consistent with endothermic peaks of PEEK and PGA [19,20]. Moreover, with increased content of HAP particles, the temperatures at which the two exothermic peaks occurred generally increased. This might have been because HAP particles would absorb heat during heating processes. Hence, more time was needed for PEEK and PGA in the composite to take in enough heat to attain their decomposition points, recalling that temperatures varied linearly with time. These results corresponded to those observed in other researches on the compound of other polymers and HAP [21,22].

### 2.4. Mechanical Properties

**Figure 5:**
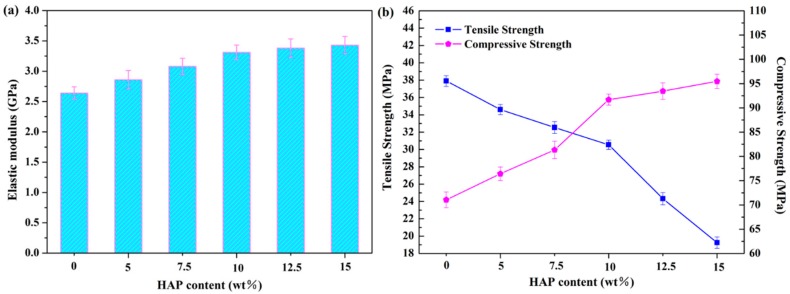
Elastic modulus, compressive strength and tensile strength of the scaffolds with different HAP contents.

The elastic modulus and compressive strength of the scaffolds increased with the HAP contents (Figure 5). The addition of 10 wt % HAP could increase elastic modulus to 3.31 GPa, being 25% higher than that of the PEEK/PGA scaffold. Moreover, compressive strength increased to 95.47 MPa with incorporation of 10 wt % HAP, being 34.5% higher than that of the PEEK/PGA scaffold. The elastic modulus and compressive strength increase with inorganic filler contents were common in the polymeric composite because of the higher stiffness of the filler [23,24,25]. Nevertheless, the tensile strength of the scaffolds decreased with HAP contents, and substantially dropped to about 24.3 MPa when the HAP content reached 12.5 wt % (Figure 5b). The severe HAP agglomeration in the polymeric matrix (see Figure 2) probably caused this strength decrease. Therefore, the optimal HAP content was 10 wt % in this study.

### 2.5. Bioactivity Evolution

**Figure 6:**
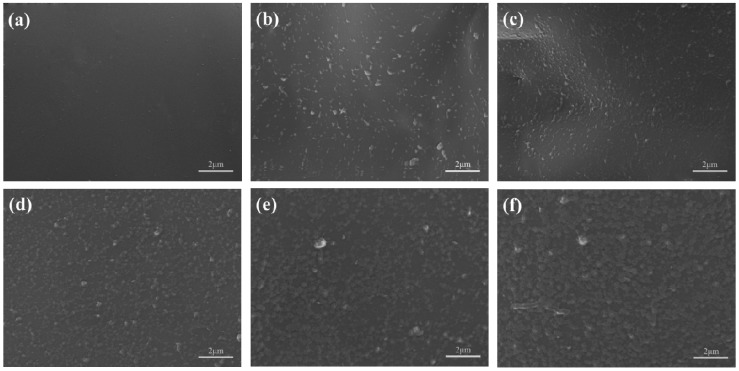
Morphologies of the scaffolds with (**a**) 0 wt %; (**b**) 5 wt %; (**c**) 7.5 wt %; (**d**) 10 wt %; (**e**) 12.5 wt %; (**f**) 15 wt % HAP after immersion in simulated body fluid (SBF) for 14 days.

The bioactivity of the scaffolds with different HAP contents was evaluated by soaking them into simulated body fluid (SBF). There was no deposition observed on the surface of the scaffolds without HAP after immersion in SBF for two weeks (Figure 6a), which confirmed that the composite of PEEK and PGA lacked apatite formability. Only a few depositions were found on the PEEK/PGA-5 wt % HAP scaffold (Figure 6b). With the increase of HAP contents, the amount of particle depositions increased. Moreover, the scaffold’s surface was overlaid by a layer of worm-like precipitates, with HAP increasing to 15 wt % (Figure 6f) after 14 days of immersing. These results indicated that the incorporation of HAP particles into the PEEK/PGA matrix would stimulate and accelerate the apatite formation, and therefore enhanced bioactive behavior of the scaffolds.

**Figure 7:**
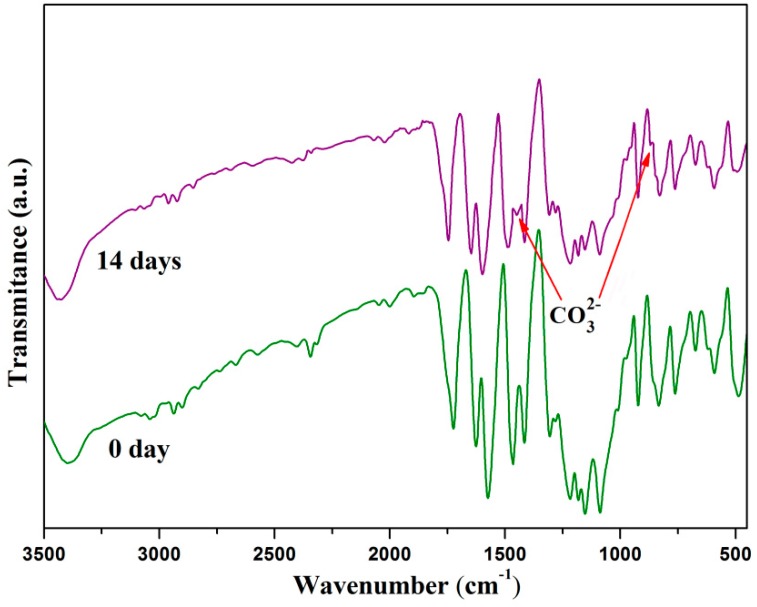
Fourier transform infrared spectrums of the scaffolds with 10 wt % HAP before and after immersing in SBF.

FTIR was used to provide more information about apatite formation on the scaffold's surface after soaking in SBF. FTIR spectrums of the PEEK/PGA-10 wt % HAP scaffolds before and after immersion in SBF for two weeks were recorded, ranging 450 cm^−1^ from to 3500 cm^−1^ (Figure 7). After 14 days of immersing, the spectrum exhibited some new and small peaks, which corresponded to the vibrational band of carbonate group. These were ν_2_ band at 879 cm^−1^, ν_3_ bands at 1462 cm^−1^. The results demonstrated that the worm-like apatite crystals on the scaffold's surface were bone-like apatite whose phosphate ions were substituted partially by carbonate ions after 14 days of immersing in SBF. Since bone mineral contained some carbonate in human body, the appearance of carbonate in composite scaffolds was beneficial [26]. As a consequence, it could increase the bioactivity of apatite.

### 2.6. In Vitro Degradation

**Figure 8:**
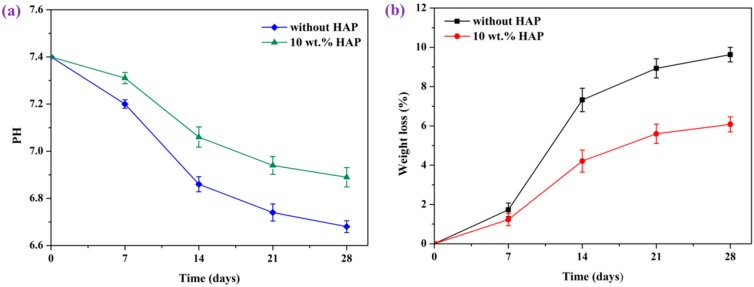
(**a**) pH value of the PEEK/PGA scaffold and PEEK/PGA-10 wt % HAP scaffold during in vitro degradation; (**b**) weight loss of the PEEK/PGA scaffold and PEEK/PGA-10 wt % HAP scaffold after immersing in PBS.

In vitro degradation behavior of the scaffolds was an important factor in tissue regeneration [27,28]. The pH changes of scaffolds during in vitro degradation in the PBS solution are shown in Figure 8a. The pH for the scaffolds declined from the oringinal value of 7.4, owing to the released acidic degradation products from PGA. At the same time, it was obvious that pH value of PEEK/PGA-10 wt % HAP scaffolds decreased much more slowly than that of the PEEK/PGA scaffolds. This could be explained thus: the degradation of PGA was shown to be comprised of hydrolysis processes, which could increase at a faster rate in the acidic atmosphere, and thus the pH changes of PEEK/PGA scaffolds exhibited the declining tendency. For the PEEK/PGA-10 wt % HAP scaffolds, –OH functional groups of HAP were able to neutralize acidic degradation product from PGA, which rendered the decrease of the pH rates less. Ameliorated acidity atmosphere could reduce the risk of inflammatory reactions during the culture of cells and tissues [29].

In-vitro degradation was also determined by the weight loss after immersing in PBS for different time (Figure 8b). Weight loss of the two scaffolds both increased gradually as the soaking time prolonged. Furthermore, weight loss of PEEK/PGA-10 wt % HAP scaffolds was a little lower than that of the PEEK/PGA scaffolds after degradation for 28 days (6.08% and 9.63%, respectively). This was because the HAP incorporated in the PEEK/PGA matrix could reduce the possibility of acidic selfcatalysis effects, and it was consistent with the measured result of pH.

### 2.7. Biocompatibility Studies

**Figure 9:**
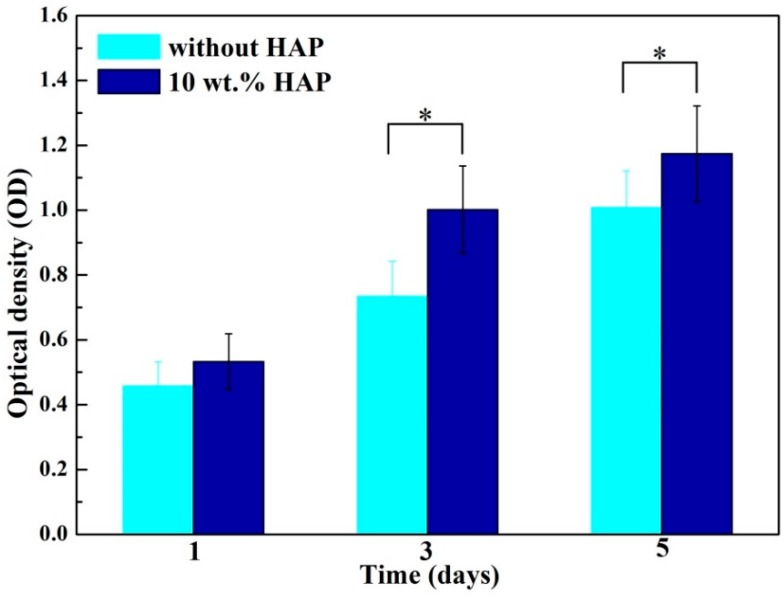
MTT assay of MG-63 cells cultured on PEEK/PGA scaffolds and PEEK/PGA-10 wt % HAP scaffolds (* *p* < 0.05).

An MTT experiment was implemented using MG-63 cells to study cell proliferation on PEEK/PGA and PEEK/PGA-10 wt % HAP scaffolds (Figure 9). As the culture times became prolonged, the cells gradually increased on the both scaffolds. In addition, the cells on PEEK/PGA-10 wt % HAP scaffolds were much more than that on PEEK/PGA scaffolds at the same incubation days (1, 3 and 5 days). The results hinted that the addition of HAP into the scaffolds was beneficial to the cells’ proliferation. Several studies had also reported that the incorporation of HAP into the polymeric matrix would improve cell proliferation [30,31].

**Figure 10:**
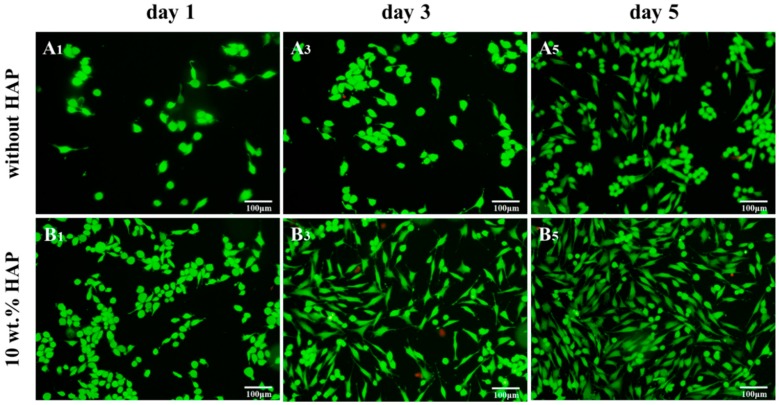
Fluorescence images of MG63 cells cultured on PEEK/PGA and PEEK/PGA-10 wt % HAP scaffolds for different periods (letters **A** and **B** correspond to the two scaffolds. Subscripts indicate the time).

Fluorescence photographs proclaimed that live MG63 cells on scaffolds appeared as green staining (Figure 10). The cells displayed ball-like morphologies at the beginning. Besides, it was obvious to observe that live cells attached well and spread with extended filopodia on PEEK/PGA-10 wt % HAP scaffolds after their incubation for three days (Figure 10B_3_), which resembled those incubated for five days on PEEK/PGA scaffolds (Figure 10A_5_). With incubation time increased to five days, cell numbers grew, filopodia of cells had further extended, and there appeared some cell fusion (Figure 10B_5_). These results further demonstrated that the scaffolds with HAP could stimulate cell proliferation and attachment. The reason was likely that the degradation of HAP might neutralize the acidic products from PGA and led to the stability of pH. Previous researches have demonstrated that a low or high pH was likely to restrained cells behavior [32]. The results of MTT and cell immunofluorescence experiments demonstrated that the scaffolds with HAP had good biocompatibility.

### 2.8. Alkaline Phosphatase (ALP) Activity

**Figure 11:**
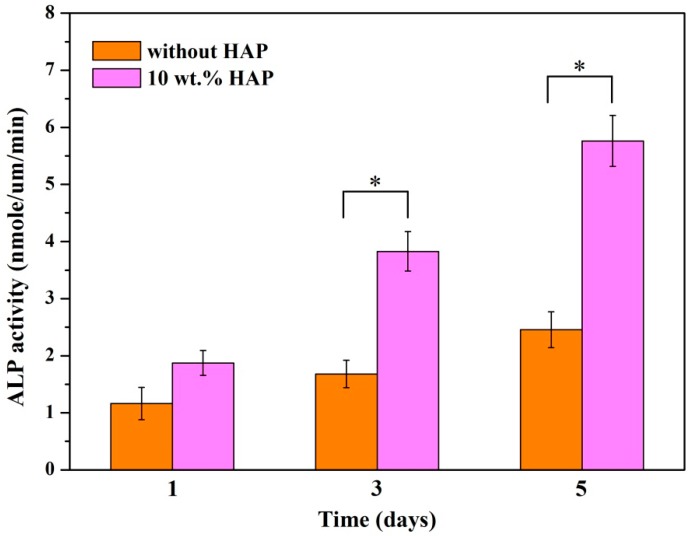
Alkaline phosphatase (ALP) activity of MG63 cells cultured on PEEK/PGA scaffolds and PEEK/PGA-10 wt % HAP scaffolds at various time points (* *p* < 0.05).

Cell differentiation was assessed by the ALP activity of MG63 cells incubated on PEEK/PGA and PEEK/PGA-10 wt % HAP scaffolds for different periods (Figure 11). The activity of ALP secreted by the MG63 cells was low on the two types of scaffolds at day one. With incubation time prolonged, the activity of ALP on the PEEK/PGA-10 wt % HAP scaffolds increased substantially. On the contrary, the activity of ALP of the cells incubated on PEEK/PGA scaffolds presented a very slight increase tendency with incubation time. This was in accordance with the previous researches using HA-containing composite scaffolds, which presented that the addition of HAP could enhance cell differentiation at an early time [33,34]. One of characteristics of HAP was its bioactive nature which could improve cell differentiation [35]. Besides, it could bind proteins and growth factors, and thus promoted cell differentiation [36]. These results implied that the scaffolds with HAP offered a favorable microenvironment for cell differentiation.

## 3. Materials and Methods

### 3.1. Materials 

Irregularly shaped PEEK (Mw: 270,000 g/mol) with a specified average particle size of 30 μm was obtained from Dongguan Guanhui Plastic Materials Co. Ltd. (Dongguan, China). PGA (Mw: 1,000,000 g/mol) was purchased from Shenzhen Polymtek Biomaterial Co. Ltd. (Shenzhen, China). Hydroxyapatite particles with rod morphology were provided by Nanjing Emperor Nano Material Co. Ltd. (Nanjing, China). Phosphate buffer solution (PBS) was supplied by Beijing Chemical Reagent Company (Beijing, China). Dulbecco’s modified Eagle’s medium (DMEM) and fetal bovine serum (FBS) were provided by Life Technologies (Carlsbad, CA, USA).

### 3.2. Preparation of Porous Scaffolds

(PEEK/PGA)-HAP composite powders were prepared as follows: PEEK powder (80 wt %) and PGA powder (20 wt %) were sonicated for 30 min in ethanol using ultrasonication, then grinded for 30 min by a variable frequency planet-type ball mill at room temperature. Whereafter, HAP powder was added to the PEEK/PGA solution. At this stage, the HAP powder was blended in proportions of 5%, 7.5%, 10%, 12.5% and 15% of total weight, respectively. The composites were ultrasonicated and grinded vigorously for 1 h to evenly disperse the HAP in the polymer solutions. After grinding, the composites were exsiccated in a drying oven.

The obtained powders were used to manufacture composite scaffolds through SLS. The SLS system comprised a CO_2_ laser, three-dimensional motion platforms, sintering platform and control system. During sintering processes, composite powders were sintered layer by layer to develop porous scaffolds. The processing parameters remained constant: laser power 2.5 W, scanning rate 400 mm/min, spot diameter 0.8 mm, scan space 3 mm and layer thickness 0.1–0.2 mm.

### 3.3. Differential Scanning Calorimetry (DSC)

The thermal behaviors of the composite scaffolds were studied employing an STA-200 differential scanning calorimeter. In short, specimens (approximately 8 mg) were enclosed in hermetic aluminum pans. Subsequently, they were heated from 30 °C to 380 °C at a constant temperature rise of 10 °C/min under the protective nitrogen atmosphere. Six specimens were tested and the melting point temperature was identified from melting curve.

### 3.4. Characterization

Scanning electron microscopy (SEM, FEI Quanta-200, FEI Co., Hillsboro, OR, USA) was operated to analyze the morphologies of the composite scaffolds. The specimens were coated by platinum layer using a JFC-1600 sputter coater (JEOL Co., Tokyo, Japan) for 200 s. SEM was performed at 12 kV to observe the features of samples. An X-ray diffractometer (XRD, D8 Advance, German Bruker Co., Karlsruhe, Germany) was carried out to characterize the phase composition of composite scaffolds. XRD diagrams were obtained in 2θ mode ranging from 10° to 80° at the scanning rate of 8°/min. Seven samples were detected and XRD diagrams were obtained in 2θ mode ranging from 10° to 80° at the scanning rate of 8°/min. The compressive strength was tested on an MTS Insight electromechanical testing machine (MTS systems Corp., Shanghai, China), and the screw speed was 0.5 mm/min. Tensile tests were determined using a WD-D1 testing machine (Shanghai Zhuoji Instruments Co., Ltd., Shanghai, China) with a crosshead speed of 5 mm/min. The strip shaped specimens were 10 × 10 × 5 mm^3^. Elastic modulus was obtained with the initial slopes of stress–strain curves. Five specimens were tested at each composition and the mean values were reported.

The porosity (*P_d_*) was evaluated using the equation:

(1) $$P_{d}=\frac{V_{p}-V_{a}}{V_{p}}\times 100\%,$$

where *V_p_* (mm^3^) is the apparent volume that was obtained based on the geometry, *V_a_* (mm^3^) is the actual volume that was estimated based on the Archimedes’ Principle, and *P_d_* (%) is the porosity. The final porosity of fabricated scaffold was the average of five test data.

### 3.5. In Vitro Biomineralization Properties

The biomineralization properties were evaluated by soaking the composite scaffolds with different HAP contents in SBF solution. This solution was prepared as the Kokubo protocol by dissolving reagent-grade chemicals into distilled water, with a similar concentration to those of human blood plasma (Table 1). It was maintained at 7.4 with Tris(hydroxymethyl)-aminomethane and 1 mol/L HCl. The six scaffolds were immersed in SBF for 2 weeks at 37 °C, and the solution were renewed every second day. After immersion, they were extracted, rinsed with ethanol and dried at 37 °C for 12 h. The microstructure and chemical groups of the deposited apatite layer on scaffolds were assessed using SEM and Fourier transform infrared (FTIR, Thermo Scientific Co., Madison, WI, USA) spectroscopy, respectively.

### 3.6. Degradation Behavior

In vitro degradation of the composite scaffolds was evaluated in PBS solution (initial pH was 7.4). Three samples were weighted (*W*_0_) for each group and subsequently immersed into the solution in incubators at 37 °C for different times (7, 14, 21, 28 days). The pH changes in PBS at each period were determined using pH meter. At the predetermined time, they were removed from the buffer, thoroughly cleaned with ethanol and desiccated to a constant weight (*W*_1_) at 50 °C for 12 h. The weight loss ratio was determined as:

(2) $$\mathrm{weight}\;\mathrm{loss}\;\left(\%\right)=\frac{W_{0}-W_{1}}{W_{0}}\times 100\%,$$

Each group was measured six times and the values were averaged.

### 3.7. Cell Culture

The biocompatibility of composite scaffolds was investigated through cell culture experiments. Before cell seeding, scaffolds were sanitized with 70% ethanol for 2 h, then sterilized under ultraviolet for 15 min and rinsed three times with PBS. Afterwards, they were placed into a 12-well plate, seeded with 20,000 MG-63 cells (Cellular Biology Institute, Shanghai, China) per well and incubated in Dulbecco’s modified eagle mediums with a 5% CO_2_ humidified incubator at 37 °C. The cells were passaged three times prior to use. Three samples were fostered for each group and the culture medium was renewed every two days. After different culture periods (1, 3 and 5 days), scaffolds were taken out from plates and cleaned in PBS. Afterwards, the cells attached on scaffolds were immobilized with 4% glutaraldehyde and dehydrated with ethanol.

Cell viability of composite scaffolds was measured by MTT assay. At preset culture time, 10 μL MTT solution was added into each scaffold and incubated at 37 °C for 3 h. Then the supernatants were discarded and 600 μL dimethyl sulphoxide (DMSO) was put into each well to dissolve intracellular formazan. Three scaffolds were incubated for each group and the absorbency at 570 nm was recorded with the microplate reader. Moreover, cell-material interaction was assayed by fluorescence technique. After cell culture, the composite scaffolds were given out from plates and purged with PBS, fixed by paraformaldehyde and permeabilized. Hereafter, cells were cleaned and preincubated with PBS. Afterwards, the cells were cultivated in 4 µm EthD-1 for 30 min. Finally, fluorescence figures were obtained by the confocal microscope (Leica Microsystem, Mannheim, Germany).

### 3.8. Alkaline Phosphatase (ALP) Assay

ALP activity was conducted to analyze the MG-63 cell differentiation for different culture days. Scaffold specimens were placed in a 24-well plate. MG-63 cell were cultured on specimens for 1, 3 and 5 days. The culture mediums were refreshed every three days. At selected time points, the adherent cells were rinsed twice in PBS, and incubated in a cell lysis buffer that contains 0.1% Triton X at 4 °C for 30 min. After that, cell supernatant was detected for ALP activity by *p*-nitrophenyl phosphate, and absorbance was determined at 405 nm. The ALP assay of MG-63 cells was performed by a Laboassay™ ALP kit (Wako Pure Chemicals, Tokyo, Japan) according to the manufacturer’s instruction. Results showed mean values of three individual tests.

### 3.9. Statistical Analysis

All data from experiments were statistically analyzed using Origin 6.0 software (Microcal Software Inc., Northhampton, MA, USA) and expressed as mean ± standard deviations. Statistical significance was identified using Student’s *t*-test, considering statistically significance at *p* < 0.05.

## 4. Conclusions

The PEEK/PGA scaffolds incorporated with HAP were manufactured using a selective laser sintering system. The addition of HAP could not only improve the bioactivity of the scaffolds, but also was beneficial to cell attachment and proliferation. In addition, it could enhance the elastic modulus of the scaffolds. Therefore, the PEEK/PGA scaffolds with HAP were more attractive for bone tissue regeneration.

## Figures and Tables

**Table 1:** Ion concentration in SBF and human blood plasma.

Ion Type	Ion Concentration (mM)						
	Na^+^	K^+^	Mg^2+^	Ca^2+^	Cl^−^	$HCO_{3}^{-}$	$HPO_{4}^{2-}$
Simulated Body fluid	142.0	5.0	1.5	2.5	148.8	4.2	1.0
Human blood plasma	142.0	5.0	1.5	2.5	103.0	17.0	1.0
